# The Treatment of Lesions at and about the Head of the Colon

**Published:** 1890-06

**Authors:** Joseph Hoffman

**Affiliations:** Philadelphia, Pa., Fellow of the American Association of Obstetricians and Gynecologists; 126 W. Diamond Street


Buffalo Medical and Surgical Journal

VOL. XXIX. JUNE, 1890. NO. 11.

### Original Communications.

### THE TREATMENT OF LESIONS AT AND ABOUT THE HEAD OF THE COLON.[1]

By JOSEPH HOFFMAN, M. D., Philadelphia, Pa.,

Fellow of the American Association of Obstetricians and Gynecologists.

HYPOTHETICAL pathology has rendered the current text-book literature on the subject of inflammations at the caput coli practically worthless. Whatever of literature there is worth reading and remembering, is scattered in the pamphlet and periodical publications of the last decade, and even yet it is not crystallized into forms that give it permanency and value to those outside the pale of the progressive teachings and the generally accepted views of to-day. There is always opposition to progress when it is in a line diametrically opposed to current ideas. In the present case, there is a border warfare between the old time medicine men, who yet hold to the view that there is a perityphlitis, a distinct lesion, outside and apart from the peritoneum, just as there is a periproctitis, without relation to it. To these, that there should be any other treatment for "perityphlitic abscess" than poultices, opium and rest, is the extreme of absurdity, while they who would resort to radical and prompt treatment surgically, are considered rashly aggressive. The subject under consideration has of late been so variously dealt with, that it is the aim of the present paper to endeavor to weed out some unessential refinements in all that relates to it, and to place it upon a basis common alike to all who may have to deal with it as a medical or surgical reality. First, then, as to the pathology of these lesions. The term perityphlitis is a misnomer; it does not obtain as an original lesion, but is a transference from a preëxisting inflammation, it being conclusively shown by post-mortem examination that

1. Read at the annual meeting of the Alumni Association, Medical Department, Niagara University, April 15, 1890.

in the great majority of cases the appendix is the primary seat of the inflammation. At the beginning of this paper, it was remarked that hypothetical pathology had rendered the current text-book literature on this subject practically worthless. At the bottom of this fact lies that egregious misconception that the appendix lay outside the peritoneal cavity, and even that the cecum itself was similarly situated. Where anatomy was so grossly at fault, pathology could not be correct, and the results of the error, far reaching for evil, have not yet obtained their limit. But while we are set right as to the original lesion in this group of affections, however complex they may ultimately become, there has been attempted a refinement in differentiating the successive steps and conditions that arise from this common cause, that to the minds of many it must appear that there is a benign and a malignant phase at once to be diagnosticated and distinguished. Herein arises the question of cecal inflammation. Does it exist apart from appendicitis? Although post-mortem proofs of such inflammations are rare, yet from the anatomical position of the cecum, the inference is certainly justifiable, that such lesions occur. It is rather a *post hoc* inference, however, than a logical deduction. It would seem impossible, nevertheless, for a pericecal inflammation to occur without the involvement of the pelvic peritoneum, since Treves has shown that there is no pericecal connective tissue, properly speaking, while he also holds the view that there is no meso-cecum. Inflammations of the cecum may occur directly from the irritation of foreign bodies. This in Fitz's investigations was found to be the only cause.

The appendix, then, a singularly useless organ, so far as we know, is the cause of most of these lesions. Anatomically considered, it is most inconstant, varying in length greatly. In the Pennsylvania Hospital Museum, there is a specimen nine inches long. When of such great length, it usually extends upwards in the abdominal cavity.

Impactions in its lumen are the cause of its inflammation, and the local or general peritonitis set up by transference or contiguity, are at the start the same, though varying in their degree, and in the definition of their boundaries, according to the extent of the original inflammation, and the location of the appendix which decides the direction in which the inflammatory products will burrow.

Given an inflammation, there is no assurance, either that it will remain quiescent, will remain pelvic, with a tendency to burrow outward, or that it may not generally infect the peritoneum. The burrowing may take place in a variety of directions. Musser has shown that it may discharge through the abdominal wall, the scrotum, into the hip-joint, through the loin into the peritoneum, and sometimes into the pleural cavity, and even into the bladder. With this showing it seems almost superfluous to urge the abandoning of refinements in the diagnosis of pathological conditions, with a symptomatology pointing, in general terms, to the existence of vermiform disease. If we are to retain the terms, perityphlitis, typhlitis and paratyphlitis, let it be only to remember that they do not exist save as the consequence of an affection whose sequelæ they are.

The diagnosis of inflammations of the appendix, or of abscess around the head of the colon, cannot be considered easy under all circumstances. In a number of cases lately reported, the operation was done with no thought of existing appendicitis—the indications being simply general—the idea being to remove a pathological mass. The appendix is often diseased when there is little or no sign that such is the case, or when whatever symptoms there are, are attributed to something else. That such is the fact is proven by a large number of general autopsies. We must agree, then, that a simple catarrhal inflammation, or even a slight impaction in the organ, without peritoneal exudation or involvement, will generally escape exact diagnosis. If we have and hold in mind the possibilities of such disease, we may suspect its presence without, however, being able to prove it. I have already referred to the rarity of primary perforating disease of the colon. This has been proven, even in chronic dysentery and tuberculosis. That there may be a colitis from fecal impaction is not disputed, but when the trouble arises from such a cause, a tumor must be present from the very inception of the retention, there is an absence of a primary inflammation, there is a history of habitual constipation, while there is a notable absence of fever. These are the cases that end in resolution, and encourage those who do not discriminate carefully to believe that they have cured a perityphlitic abscess, and that after all the condition is not nearly so bad as it is represented. That fecal impaction may cause a perforating appendicitis, cannot be questioned, as the following history from a case under my observation well illustrates:

I was called in consultation to see a negro, aged 25. He had a history of great irregularity of the bowels; some general discomfort, which finally eventuated in the attack from which he was suffering when I saw him. He had a well defined tumor in the right inguinal region, and extending upward as a lumbar fulness. The mass was painful under pressure, and examination, per rectum, showed it to be boggy. The fever was, however, not marked, though there was evidently general prostration from some cause. He had been treated with quinine and anodynes, which, however, did not permanently remove the pain. I advised free purgation, and the following day went prepared to operate in case the symptoms were more urgent. I found him entirely relieved of his pain, sceptical as to my opinion as to the serious nature of his illness, and scarcely willing to be touched at all. The tumor had disappeared, and he was left to the treatment of his physician, who also quietly enjoyed the rout of radical surgery.

Two months later I enquired of the same physician as to the condition of his patient. "Dead," was the answer. He went to Baltimore, had a recurrent attack, and this was the end. What form the attack assumed I regret my inability to learn. Here was a man in whom, in all probability, fecal impaction was the origin of an appendicitis perforans, that finally, under repeated attacks of constipation, resulted in general peritonitis. Another case of undoubtedly the same nature is a living illustration of this same pathology:

A young man, aged 22, came under my care with a primary attack of like nature. There was pain under pressure, no especial rise in temperature, and a fulness in the right cecal region. I purged him freely, applying turpentine stupes over the site of the swelling at the same time. The evacuation of the bowels was followed by a complete relief from all pain. There has been no recurrence of the disease, though three years have elapsed, a fact taken in connection with all other points in the history, justifies the diagnosis of colitis dependent upon stercoral impaction.

That there was in this case, or can be any involvement of the so-called pericecal tissue, I do not believe. For such a condition, primary perforation is necessary, or a breaking down of the walls of the cecum from the pressure of impacted fecal matter. Now, while these cases may go on toward resolution uninterruptedly, a certain proportion of them end in suppuration and abscess. Such cases can only be treated successfully by operation. The use of an exploring needle to determine the presence of pus, I do not believe can be justified under any circumstances. At best, the aspiration only makes plain what is logically present without its use, while if it reveals the presence of pus, it helps us not one jot in the treatment of the condition. Apart from this, if the puncture is negative, it may lull into a sense of false security, besides doing harm *per se*.

The initial history of a perforating appendicitis is altogether different from that above described; its oncoming is sudden. The primary lesion, which may be considered to have been catarrhal, is impossible of diagnosis, has finally lead to an inflammatory thickening of the lining mucosa of the appendix, narrowing its calibre, and preventing the escape of any fecal matter, or foreign body accidentally contained. This is the initiation of a condition that results in perforation. The symptoms are fulminant in character,—intense pain, rendering the slightest movement impossible. Collapse often speedily occurs, followed speedily by death. These are, of course, extreme cases. If the patient is so fortunate as to survive the first shock of the disease, peritonitis is soon a complication of affairs, and may be limited by adhesions or rapidly become general. In the latter event it is almost certain to be fatal. Here, as in the other class, operation is the only hope for the patient.

Again the attack may assume a sub-acute condition that is misleading in the extreme. The pain may in great part subside, the fever abate, and the patient seem on the road to early recovery. Abdominal percussion may give no clue to the deposit at the seat of the perforation. Hence palpation and percussion externally are not to be relied upon. Examination by the rectum will often reveal a fulness or even tenderness, otherwise not to be detected. This last feature is to be explained by the frequent location of the appendix under the cecum. This, of course, interferes with the use of the aspirating needle with the expectation that it will have any chance of helping us to a diagnosis. Apart from the more serious symptoms, that of vomiting is to be considered. It is worthy of especial comment, that in the lesion, by all odds the most serious, it is not so marked as in cases of cecal impaction or simple obstruction. As to the differential diagnosis of these affections with other abdominal diseases, it is to be impressed upon the mind of the practical physician and surgeon, that while a variety of conditions may suggest themselves as possible, *all or the most of such conditions are to be remedied by operative interference, not by medication.* This is true, almost without exception.

If we have a concealed hernia to deal with, the remedy is operation; so with intussusception, strangulation of the bowel, or obstruction. In all other affections, where pain is present without other symptoms, the history offers for the most part distinctive symptomatology, and this being absent where there are symptoms of pus, it is safer to confirm the diagnosis by exploratory incision than to hope for resolution. In women, added to the conditions noted above, we may have also the various surgical affections peculiar to the sex. Or, we may have disease of the appendix complicated with ovarian disease, either abscess or cyst. It is a common condition in which inflammatory adhesions are found to exist between the appendix and ovary. The same is true in the presence of extra-uterine pregnancy of the right side. But in the presence of the difficulty of such complications, we are again to escape the dilemma by resorting to early operation, following the now accepted truism, that early operation saves lives in all these affections, while delay loses them.

The logic of early operation in appendicitis, is that even if the urgent symptoms subside for a time, they are prone to return, each succeeding attack being worse than its predecessor, and finally from some unusual exertion or accident, a rupture occurs in the preëxisting adhesions, and a general peritonitis is started up and speedily ends fatally. Now, when this is true, delay at the best only averts a present operation for a future calamity, insuring death for the patient, and failure for the surgeon who is called on in the emergency.

It is not to be forgotten in the diagnosis of perforating appendicitis, that sex is an important factor. The proportion of men to women so attacked is as four to one. Statistics also show that age is always to be considered in making a diagnosis. Young or youthful individuals seem more prone to such inflammations than the old or middle aged. As an introduction to a consideration of the treatment of this or allied affections, I wish here to introduce an analysis of the more leading aspects of such cases, as determined statistically. For this purpose I shall use the tables of Fitz. Two-thirds of the cases tabulated by him were of unquestioned abscess. Only one-fourth of all cases may be expected to undergo resolution. Recurrency is so frequent that resolution would seem to indicate not a chance of cure, but only of temporary palliation of symptoms. Again in sixty out of 176 cases of perforating disease of the appendix, thirty-four per cent. died in the first five days, and in seventy-three cases of general abdominal pain, peritonitis occurred as early as the fifth day in fifty-four cases, or seventy-four per cent. Hence the time for operation must be set early, to escape the danger of the complication of peritonitis, or even the general exhaustion of the patient.

The question of operation in lesions of the kind we have been considering, strange as it may seem, is one still far from being settled. Physicians—purely such—are often prone to dispute its necessity, primarily, at all. They lay their trust in opium, and are content to let their science begin and their art end with the fabled splinting of the bowels. Surgeons without an abdominal experience, from a purely general standpoint, lay down principles and methods of procedure with which the abdominal surgeon cannot for a moment agree. Agreeing upon the necessity of operation, in a majority of cases, surgeons of undisputed ability disagree as to where the incision should be made, and as to the time of operation.

With such questions still unsettled, or in dispute, which is worse, (for there are sufficient data at hand to decide all such points for practical purposes and get results,) is it any wonder that the general practitioner is unsettled as to how he shall proceed? The time, it would seem, is ripe for some general conclusion, such as is held and subscribed to in most other serious conditions.

Though such a consummation is devoutly to be hoped for, it is not to be reached by dogma, or one man's personal experience. It must rather be sought after the manner of the collective investigation of disease, by differentiating personal experiences, striking out the infinitesimals of prejudice, eliminating common differences, and retaining the valuable remainders.

From what, then, are we to hope the best results in the treatment of these conditions? What is the philosophy of the administration of opium; what does it accomplish; what does it prevent? Primarily, opium relieves pain. This effect *per se* is desirable, but coupled with its other action, in the presence of real lesion, that of masking symptoms which are danger signals and give us data for active interference, its use is negatived. In any condition where irritation is present, rather than inflammation, it is not needed, and its anodyne effect is equaled by gentle purgation, which need be none the less thorough, because mild. If there is present in the appendix an irritating body, and this it is to be remembered is oftenest the condition of affairs, opium can do no possible good, neither can any other medication which favors intestinal engorgement. Opium, moreover, paralyzes the intestines, favors the accumulation of gases, thereby increasing constantly the abdominal tension, and rendering the splinting of the bowels theoretical, not real. Before there are symptoms of pus, and it is to be remembered this is often an open question up to the time of operation, the pain is likely to subside with free purgation, while an after-treatment of rest and little more than occasional use of anodynes is by all odds safer than a previous routine medication with opium. Now, what is the logic of saline or calomel purgation? It is to be remembered here, that in the presence of actual impaction of the appendix, and consequent tendency to a primary local peritonitis, purgation is to be advised as a prelude to actual removal of the appendix, not as a curative agent. When the intestinal canal is clear of its contents, without previous paralyzing medication, it preserves its natural contractility, and resists any tendency to tympany, so generally present after the use of opium. Further, purgation, while it drains the intestinal canal, acts as a natural derivative, imitating actively the process which is hoped for by the alterative administration of calomel. Where there is pus in the pelvis beyond doubt, the lesson of abdominal surgery is, that previous purgation immediately antecedent to the operation is of positive advantage. Where there is general peritonitis, immediate operation with enucleation of the offending body with immediate after-drenching of the peritoneal cavity, cannot be too strongly urged. No operation that does not remove the appendix can be regarded as a safe procedure; for leaving *it* we leave all that renders operation necessary. The exception to this is where a patient is found *in extremis*. The operation must then be done to save life; incision and drainage, with this end in view, are all that is justifiable. It is by all odds the best surgery, in the light of recurrence, and the fatality of general peritonitis, to operate early upon suspicious cases. It is to be regarded in abdominal surgery as proven, that pus anywhere within the abdominal cavity is more dangerous than operation to remove it. The argument to wait for adhesions before operation, is fallacious. While waiting for adhesions, we are just as liable to wait for a general peritonitis.

Early operation before adhesions are formed will enable us more easily to reach the diseased part, involve less handling of the intestines, shorten the operation, all of which are factors favoring the recovery of the patient. Having agreed upon operation, what as to the incision? Here we are directed on the one hand to make it over the appendix region. On the other hand we are directed where there are symptoms of general peritonitis that the incision should be median, or combined with the lateral, it being held that this affords the best facility for drainage. Let us look for a moment at this from the standpoint of gynecology. Given a pyosalpinx with all its complexity of adhesions, the bottom of the pelvis, omentum, intestine, and appendix itself, where does the operator make the incision? In the median line. So in extra-uterine pregnancy, than which, when ruptured, no disease can reveal more extensive adhesions. In pyosalpinx, too, abscess cavities, with necrosed dêbris and virulent pus are as a rule present, but the incision is median. Now, if in these conditions, drainage and enucleation are possible, why should it not be in disease of the appendix? The argument that the peritoneum is unnecessarily opened by the median incision, has no foundation in fact. If we break up adhesions, remove dêbris, and the appendix, we must open the peritoneum, and the opening in the middle line enables us far better to wash out inflammatory remnants. As an illustrative case I will quote the following, which practically answers all of the above objections showing that they are purely theoretical, and clinically illustrating many of the points referred to throughout this paper:

On first seeing the patient, Mrs. B., married, with three children, I found her with a pulse of 128, correspondingly high temperature, and unable to stir in bed without extreme pain. Careful questioning gave me the information only that eight days previous she had slipped from a chair, and caused thereby severe pain in the right iliac region. This had continued up to the time I was called, when she was compelled to take to her bed. The existence of her injury, according to her own statement, was limited to a little more than a week, though, in connection with this, she mentioned the occurrence, previous to her menstruation, of an ill-smelling vaginal discharge. Examination, per vagina, discovered the presence of a growth alongside of the uterus, the touch of which gave her much pain. The rectum was empty, she having had a dysenteric attack the day previous. Touch outside was so painful that I did not attempt surface examination. The pain in her back and right leg was intense. I decided that operation was necessary, and called Dr. J. Price in consultation, to confirm my opinion. We agreed that operation was necessary, but, examining under ether, were somewhat uncertain as to the condition.

Operation being decided upon, an opening was made in the median line, and exploration made. The cecal portion of the intestine was found matted down, and I freed it after much difficulty. The appendix was almost completely buried in the pelvic tissue of the iliac fossa, and the temptation was big to tie it off piece by piece, though it was afterward enucleated by persistent effort, and contained a fecal mass as the source of the inflammation. The cecal portion of the bowel was almost gangrenous in spots, and nearly ulcerated through.

Surrounding this portion was a quantity of stinking pus, about two ounces. The pelvis was carefully washed out, no antiseptics being used, a drainage-tube introduced into the cul-de-sac, and a rubber tube led from the fossa, through the incision, which was closed with seven deep and superficial sutures.

Nothing was done with the bowel, save to cleanse it.

The bowels were at first opened by enema, and after a quantity of scybala was discharged, calomel, in doses of one-sixth of a grain, was given, to clear the tongue and to relieve bilious vomiting. The patient made an uninterrupted recovery, all the stitches being removed, as well as the tubes, by about the tenth day.

The patient, at the twenty-fourth day, sat up, entirely free from pain. A curious feature of the case is, that after removal of the offending appendix, the patient, after three days, remarked she had never been so free from pain for *two years*, then going on to give an exact history of her trouble, all of which pointed to appendicitis. Her pain had become so much a part of her, that she did not seem to recognize it as foreign.

The points principally to which attention may be called are the closing of the incision and the location of the same. Although central, drainage was perfect, and, though sutured, it promptly healed; showing, I think, that dogma, both as to location and allowing the incision to remain open, is not wise.

As, in this case, the central incision enabled me to remove at the same time an ovarian hematoma, otherwise out of reach; and as drainage was perfectly obtained, these points, for such operation, are worthy of special consideration.

So far as the treatment of the intestine is concerned, this case cannot be taken as an index. The treatment must depend on the nature and extent of the lesion. It may vary from the extreme simplicity of thoroughly cleansing, to resection, or intestinal anastomosis. With such possibilities, it is evident that none but a surgeon should attempt to deal with it. The appendix, when alone diseased, may either be ligated and cut, and carefully cleansed, or, having been cut, inverted into the bowel, as suggested by Morton, and the peritoneal edges of the depression so produced, stitched together.

In three other cases lately come under my notice, the central incision was also used, for the reason that appendical disease was not positively made out, and the results in every way confirm the entire practicability of median laparatomy in the operation. Let me repeat and emphasize, that where general drenching of the peritoneum is desirable, and in the presence of pus it is always so, the median incision is no matter of experiment, both for thorough drainage and general exploration. The question of chemical antisepsis I shall not enter upon here, only to say, that in a long series of cases where pus and débris have been removed, both in my own work and that of my operating acquaintances of like opinion, there has been no need of any antiseptic whatever, and that the results have been uniformly good. The post operative treatment of these cases does not differ in any respect from that of other abdominal lesions. In general words, it can be summed up: the bowels should be kept easily open, diet should be light, and opium should be avoided, unless it is easily apparent that the restlessness of the patient will cause greater danger than that produced by the anodyne.

In summing up the treatment of the real surgical lesion of this region, which can only be considered plural when sequelæ are mistaken for primary affections, I conclude:

1. That it should in every instance be considered as a surgical, not a medical, lesion, and that medicines have no place in its treatment, save in the preparation for surgical interference. The cases that recover without recurrence are, in all probability, cases of stercoral impaction, antecedent to suppuration.

2. That the use of opium as a primary treatment is in any case, and at all times questionable, from a logical standpoint, and that any case that recovers under it, recovers not by reason of it, but in spite of it.

3. That the use of purgatives, preceded by a glycerine enema, is at once the simplest and most logical way of reducing congestion and fecal engorgement, and that cases in which it is feared that such treatment will excite either general or local peritonitis, by producing perforation, are already within the pale of operative surgery, and can in no way argue against the use of purgatives, or invalidate the logical claims for their administration.

4. That in view of the insidious nature of the disease, the frequent impossibility of exact diagnosis, and the early date at which cases apparently simple, may become fatal in character, prompt operation affords the best assurance of recovery, especially in the light of the frequent recurrence, for which, in the language of Fitz, "the eventual treatment by laparatomy, in all events, is generally indispensable."

5. That the line of incision cannot be arbitrarily located, and that for general purposes the median will answer quite as well, and in special cases better, than the lateral; and that in women, owing to the infrequency of the affection, the median incision is generally advisable.. In cases in which it is evident the inflammatory process is localized, with plain tendency to pointing in the groin, incision directly over the mass is preferable.

6. That the appendix, when operation is necessary, should always be removed (barring operation *in extremis*), and that while in exceptional cases the extra-peritoneal treatment may be possible as a result of very limited local inflammation and adhesions, this condition of affairs cannot be accepted as common, and should not be taken for granted.

126 W. Diamond Street.

